# Integrated Metagenomic Assessment of Multiple Pre-harvest Control Points on Lettuce Resistomes at Field-Scale

**DOI:** 10.3389/fmicb.2021.683410

**Published:** 2021-07-09

**Authors:** Lauren Wind, Ishi Keenum, Suraj Gupta, Partha Ray, Katharine Knowlton, Monica Ponder, W. Cully Hession, Amy Pruden, Leigh-Anne Krometis

**Affiliations:** ^1^Department of Biological Systems Engineering, Virginia Tech, Blacksburg, VA, United States; ^2^Department of Civil and Environmental Engineering, Virginia Tech, Blacksburg, VA, United States; ^3^The Interdisciplinary PhD Program in Genetics, Bioinformatics, and Computational Biology, Virginia Tech, Blacksburg, VA, United States; ^4^Department of Dairy Science, Virginia Tech, Blacksburg, VA, United States; ^5^Department of Animal Sciences, University of Reading, Reading, United Kingdom; ^6^Department of Food Science and Technology, Virginia Tech, Blacksburg, VA, United States

**Keywords:** antibiotic resistance genes, antibiotic resistome, antimicrobial resistance, agriculture, manure, lettuce, metagenomics, next-generation sequence

## Abstract

An integrated understanding of factors influencing the occurrence, distribution, and fate of antibiotic resistance genes (ARGs) in vegetable production systems is needed to inform the design and development of strategies for mitigating the potential for antibiotic resistance propagation in the food chain. The goal of the present study was to holistically track antibiotic resistance and associated microbiomes at three distinct pre-harvest control points in an agroecosystem in order to identify the potential impacts of key agricultural management strategies. Samples were collected over the course of a single growing season (67 days) from field-scale plots amended with various organic and inorganic amendments at agronomic rates. Dairy-derived manure and compost amendment samples (*n* = 14), soil samples (*n* = 27), and lettuce samples (*n* = 12) were analyzed *via* shotgun metagenomics to assess multiple pre-harvest factors as hypothetical control points that shape lettuce resistomes. Pre-harvest factors of interest included manure collection during/post antibiotic use, manure composting, and soil amended with organic (stockpiled manure/compost) versus chemical fertilizer. Microbial community resistome and taxonomic compositions were unique from amendment to soil to lettuce surface according to dissimilarity analysis. The highest resistome alpha diversity (i.e., unique ARGs, *n* = 642) was detected in amendment samples prior to soil application, while the composted manure had the lowest total ARG relative abundance (i.e., 16S rRNA gene-normalized). Regardless of amendment type, soils acted as an apparent ecological buffer, i.e., soil resistome and taxonomic profiles returned to background conditions 67 d-post amendment application. Effects of amendment conditions surprisingly re-emerged in lettuce phyllosphere resistomes, with the highest total ARG relative abundances recovered on the surface of lettuce plants grown in organically-fertilized soils (i.e., compost- and manure-amended soils). Co-occurrence analysis identified 55 unique ARGs found both in the soil amendments and on lettuce surfaces. Among these, *arnA* and *pmrF* were the most abundant ARGs co-occurring with mobile genetic elements (MGE). Other prominent ARG-MGE co-occurrences throughout this pre-harvest lettuce production chain included: *Tet*M to transposon (*Clostridiodies difficile*) in the manure amendment and *Tri*C to plasmid (*Ralstonia solanacearum*) on the lettuce surfaces. This suggests that, even with imposing manure management and post-amendment wait periods in agricultural systems, ARGs originating from manure can still be found on crop surfaces. This study demonstrates a comprehensive approach to identifying key control points for the propagation of ARGs in vegetable production systems, identifying potential ARG-MGE combinations that could inform future surveillance. The findings suggest that additional pre-harvest and potentially post-harvest interventions may be warranted to minimize risk of propagating antibiotic resistance in the food chain.

## Introduction

Antibiotic resistance is recognized as a critical threat to human health, food security, and global development (O’Neill, 2016). In 2015, the WHO strongly encouraged the development of holistic action plans to preserve drug efficacy for usage in humans and animals (WHO, 2017). Antibiotic use in agriculture has especially been scrutinized, given that the majority of antibiotics used world-wide is devoted to livestock production. The use of antibiotics as growth promoters on EU farms was banned in 2006 (European Commission, 2005) and more recently (2017) on United States farms as part of the Veterinary Feed Directive (final rule #213), which requires veterinary authorization and oversight, rather than simple over-the-counter distribution, for the use of antimicrobials in livestock (FDA, 2013, 2017, 2018). Despite this progress in antibiotic stewardship, it is worth noting that antibiotic resistance genes (ARGs) and antibiotic resistant bacteria (ARB) are still regularly recovered from EU and United States livestock herds and the United States and some EU countries remain the top users of antibiotics for livestock (Aarestrup et al., 2001; Van Boeckel et al., 2015; Elliott et al., 2017). In addition, antibiotics are widely used to promote livestock growth in nations beyond the United States and EU, with much less regulatory oversight. As the need for food and agricultural products (e.g., crops, livestock, and fiber) increases to meet the demands of a growing global population (FAO, 2020), there is concern that the emergence and dissemination of environmentally-linked antibiotic resistant infections could intensify, especially in nations that have not yet adopted environmental antibiotic resistance mitigation policies (Van Boeckel et al., 2015). Knowledge is particularly lacking with respect to how use of livestock manure-derived soil amendments in vegetable production systems could influence carriage of antibiotic resistance in the food-chain.

A variety of agricultural best management practices are employed to prevent contamination of consumer products with pathogens (e.g., manure management, post-harvest washing), but these practices were generally developed to be effective against standard sentinels of disease risk (e.g., coliforms, *Salmonella* spp.), rather than for the control of antibiotic resistance. For example, the United States Food and Drug Administration (FDA) Food Safety and Modernization Act (FSMA) recommends composting manure prior to amending soil as a best management practice to decrease the transfer of pathogens to crops (FDA, 2015). Previous research emphasizes that composting can effectively reduce fecal indicator organisms, bacterial pathogens, and up to 100% of detected antibiotics (Storteboom et al., 2007; Wu et al., 2010; Ray et al., 2017; Zhang M. et al., 2019). However, observed reductions of pathogens and/or indicator bacteria do not necessarily ensure a subsequent decrease in ARBs and/or ARGs (Storteboom et al., 2007; Edrington et al., 2009; Xu et al., 2019). The potential for ARGs linked to mobile genetic elements (MGEs) to persist and propagate in soil amendments is especially of concern, as MGEs can facilitation ARG sharing and amplification among pathogenic and non-pathogenic ARBs (Partridge et al., 2018). In particular, class 1 integrons have been identified as key markers of anthropogenic sources of ARGs and their potential to spread across species (Gillings et al., 2015). Other MGEs, such as transposases, have been linked to HGT of ARGs through co-occurrence analysis of agricultural soil using high throughput quantitative polymerase chain reaction (HT-qPCR) (Xie et al., 2018).

Tracking movement of ARGs through environmental systems is complicated due to inherent microbial diversity (Finley et al., 2013; Graham et al., 2019) and the lack of comprehensive, or consistent, analytical strategies. Multiple quantification methods are used to evaluate antibiotic resistance in agroecosystems (culture-, molecular-, etc.) and, as a consequence, analytical endpoints are not directly comparable (Davies and Davies, 2010; Rocha et al., 2019, 2020; Pelley, 2020; Suttner et al., 2020; Wind et al., 2020). Given the biological complexity inherent in the acquisition, amplification, and mobilization of ARGs, the concept of the “resistome,” i.e., all ARGs carried across a microbial community, is often useful in assessing resistance potential (Wright, 2007). Shotgun metagenomic sequencing is an attractive approach for avoiding biases of culture- and qPCR-based methods and capturing the full range of genes, including ARGs, MGEs, metal resistance genes, and taxonomic markers, across a bacterial community.

A handful of studies to date have begun to apply metagenomic sequencing toward characterizing the influence of pre-harvest factors on amendment (Gou et al., 2018; Zhang et al., 2020), soil (Fang et al., 2015; Chen et al., 2019; Macedo et al., 2021), and vegetable (Guron et al., 2019) resistomes, but most studies only examine such factors in isolation. Gou et al. (2018) observed that composting livestock-manure decreased resistome diversity as compared to raw (source) manure prior to land application. However, examinations of soils amended with either inorganic or organic-derived fertilizers indicates that, although there may be an initial increase in some measures of resistance, agricultural soil resistomes generally return to background levels after a single growing season (Gou et al., 2018; Han et al., 2018; Chen et al., 2019; Zhang Y. J et al., 2019). In contrast, after 25-years of repeated pig manure fertilization to agricultural soils in Hunan Province, China, Xie et al. (2018) did observe that organic fertilization altered abundance and diversity of soil resistomes compared to control and inorganically fertilized soils. Interestingly, there was no observed difference in taxonomic α-diversity (i.e., total unique operational taxonomic units) between the different soil treatments (Xie et al., 2018), suggesting ARG mobilization might have contributed to a disconnect between the resistome and microbial community composition. Examinations of interactions between agricultural soils and the surfaces of crops *via* metagenomics have been limited to a single growing season, suggesting that soil amendment type can affect crop surface resistome compositions, e.g., specific drug classes and specific ARGs (Tien et al., 2017; Guron et al., 2019). For example, in a greenhouse study, Guron et al. (2019) observed that lettuce grown in soils amended with raw manure carried 2.3 and 11.1 times more aminoglycoside- and triclosan-associated ARGs on their surfaces as compared to those grown in soils amended with composted manure. Field-scale examination of the fate and transport of ARGs through agroecosystems, from manure collection and management through soil amendment and crop harvest, is essential in order identify critical control points that represent the greatest risk of antibiotic resistance spread and further refine management practices as necessary (Bengtsson-Palme, 2017; Vikesland et al., 2017; Larsson et al., 2018).

Demand for livestock-origin organic fertilizer (e.g., manure, slurry, and compost) is anticipated to continue to increase in order to support increasing global agricultural demands (Byrnes and Bumb, 2017). The aim of this study was to perform an integrated assessment of the influence of multiple preharvest factors as hypothetical critical control points for the control of lettuce resistomes at field-scale. Potential control points included: antibiotic use in cows generating manure-derived (i.e., organic) fertilizers, composting versus stockpiling manure, and amendment of soil with organic versus inorganic fertilizer. Shotgun metagenomic sequencing analysis served to characterize: (1) composition of resistomes (ARG abundance and diversity); (2) ARG-MGE co-occurrences; (3) ARG linkages with key taxonomic indicators, and (4) relative potential for ARGs to mobilize to pathogens (i.e., “resistome risk”) across amendments, amended soils, and lettuce phyllosphere samples. The integrated analysis carried out in this study provides a means to simultaneously compare and optimize the roles of various on-farm management practices for mitigating the potential for antibiotic resistance to spread through the food chain.

## Materials and Methods

The aims of this study were achieved through integrated analysis of previously published archival soil and lettuce phyllosphere metagenomic sequencing datasets described in Wind et al. (2020) and Fogler et al. (2019), as well as metagenomic sequencing of amendments carried out specifically for the purpose of this study. The amendment, soil and crop surface shotgun metagenomic sequences used in this study are publicly available (NCBI BioProject: PRJNA506850). The broader experimental design is described in the following sections.

### Generation of Manure, Composting, and Stockpiling

The methods for cattle selection and manure collection were described previously (Ray et al., 2017; Wind et al., 2018). Briefly, healthy dairy cows (*n* = 10) selected for their similar body weights, milk yield, and with no history of antibiotic treatment were treated with pirlimycin or cephapirin according to label recommendations. Beginning on 3 days following antibiotic administration, all manure (mixed feces and urine) produced was collected for six consecutive days to generate the “antibiotic manure.” To generate “control manures,” manure was collected in the same fashion from similar cows receiving no antibiotics. Antibiotic manure was subject to stockpiling [stacked outdoors in large metal bins (6 m × 2.4 m × 1.2 m)] and both antibiotic and control manures were composted in piles (5.8 m × 0.9 m × 0.8 m) in similar bins and aerated *via* a perforated polyvinyl chloride (PVC) pipe system and air pump at the bottom of the bin. Stockpiled manure and compost samples were collected at two time points: initial compost/stockpile mixture (0 day) and at the time of compost completion (63 days, Supplementary Figure 1). Thermophilic conditions (US FDA FSMA-recommended temperature of > 55°C for at least 3 days) was reached in both control and antibiotic composts on day 2 (FDA, 2015). Samples were collected from multiple locations are various depths of the manure and compost piles (3–4 m^3^) using a soil probe.

### Field Study Conditions and Lettuce Cultivation

Lettuce (*lactuca sativa*) was cultivated during the 2016 growing season (March–July) at the Virginia Tech Urban Horticulture Center (UHC) in Blacksburg, VA, United States on soil categorized as a Remus fine sandy loam and documented to have remained fallow for at least a decade. Lettuce was studied as it a popular leafy crop that is often eaten raw in the United States and has been the focus of recent foodborne illness outbreaks (CDC, 2021). Individually-bordered soil plots (3 m × 3 m) were constructed as described in Wind et al. (2018) to compare lettuce cultivation conditions in triplicate with two organic soil amendments: stockpiled antibiotic manure and antibiotic compost. All organic amendments were applied at appropriate agronomic rates (6.72 Mg ha^–1^; Day 0), and supplemental inorganic fertilizer was added to reach the required nutritional content for optimal lettuce growth. Three additional plots received inorganic fertilizer (N-P-K) only, at rates recommended for the optimal growth of lettuce (140-112-112 kg ha^–1^; Virginia Cooperative Extension, 2015) and three more plots served as a no amendment control (Supplementary Figure 1). Lettuce was grown in a mix of peat and perlite for 8 weeks, with inorganic fertilizer to avoid exogenous ARB or ARG contamination, and transplanted to the plots 30 days after the amendments were applied. The no amendment control plots were not subject to lettuce cultivation due to insufficient nutrients for crop growth. Further study details, including descriptions of plot construction, field preparation, lettuce cultivation, and patterns of culturable fecal ARB, heavy metals, and metal resistance genes in soils (Wind et al., 2018, 2020), release of targeted ARGs quantified *via* qPCR in surface runoff during natural storm events (Jacobs et al., 2019), and recovery of ARGs by qPCR from harvested vegetables (Fogler et al., 2019) are available in prior reports.

Soil samples were collected at three time points relative to amendment application: background soil conditions (−1 day), amendment application (0 days), and lettuce harvest (67 days, Supplementary Figure 1). In-depth description of soil collection and processing are available in Wind et al. (2020). In brief, at each sampling time, four 2-cm soil cores from a 5-cm depth were collected from each soil plot at a randomly generated location and homogenized into one sample on site. Lettuce plants were collected from each plot at the time of harvest (67 days, Supplementary Figure 1) and immediately processed to recover surficial microbes and their DNA from the phyllosphere (Fogler et al., 2019).

### DNA Extraction

Amendment and soil samples were stored at −80°C until DNA extraction occurred, whereas lettuce sample DNA was extracted immediately upon harvest from the field (<2 h). DNA was extracted from 0.5 g compost or manure samples of the homogenized amendment samples using the FASTDNA SPIN Kit for Soil (MP Biomedicals, Solon, OH, United States) which followed the same extraction methods used previously described for this soil (Wind et al., 2020). Inhibition was minimized using the ZYMO PCR OneStep PCR Inhibitor Removal Kit (ZYMO Research, Irvine, CA, United States). To obtain microbial DNA from lettuce, leaves were washed in a sterile 0.1% peptone and 0.1% Tween solution and hand massaged to remove surficial phyllosphere bacteria, while avoiding release of plant DNA (Fogler et al., 2019). The subsequent lettuce wash diluent was filtered through 0.22-μm filters (EMD Millipore, Merck Group, Darmstadt, Germany), which were subject to the same DNA extraction and clean-up procedures described above for manure, compost, and soil samples. All DNA extracts were stored at −80°C for up 24 months prior to subsequent metagenomic analyses.

### Shotgun Metagenomic Sequencing Analysis

Amendment (*n* = 14), soil (*n* = 27), and lettuce (*n* = 12) DNA extracts were subject to shotgun metagenomic sequencing (Supplementary Figure 1 and Supplementary Table 1). For most of the treatment and time factors, samples were sequenced in triplicate. The amendments prior to manure management (0 day) and the soils prior to amendment (−1 day) were considered homogenous across the treatments and were grouped together as the control replicates. Samples and corresponding replicates were selected for sequencing depending on treatment and time effects of interest. Details on samples subject to metagenomic sequencing are reported in Supplementary Table 1.

Amendment metagenomes were prepared using the Nextera XT library prep (Illumina, San Diego, CA, United States) and sequenced on an Illumina NextSeq 500 (2 bp × 75 bp, paired-end) at the Scripps Center for Computational Biology and Bioinformatics (La Jolla, CA, United States), which is the same preparation and sequencing methods of the amended soils. The lettuce samples were prepared using the Accel-NGS 2S DNA kit (SwiftBio, Ann Arbor, MI, United States) and sequenced on an Illumina HiSeq 2500 (2 bp × 100 bp, paired-end) at the Biocomplexity Institute of Virginia Tech (Blacksburg, VA, United States). Complete sequencing preparation details are available in Wind et al. (2020) and Fogler et al. (2019). Raw reads and associated metadata were uploaded to the NCBI BioProject: PRJNA506850 for each pre-harvest critical control point of interest.

#### ARG Annotation and Normalization

Paired-end raw sequences were processed using MetaStorm using the read-matching pipeline (Arango-Argoty et al., 2016). On average, each amendment, soil, and lettuce sample contained 24,650,830, 23,610,275, and 12,292,992 raw reads (see Supplementary Table 2 for all metagenomic sample sequencing metrics), respectively, after TRIMMOMATIC quality filtering (< 0.16% reads dropped *via* filtering, Bolger et al., 2014). ARGs were annotated against the Comprehensive Antibiotic Resistance Database (CARD v2.0.1; Jia et al., 2017) with at least 80% identity using the read match sequence pipeline of MetaStorm. Known housekeeping genes were removed prior to gene abundance analysis (Wind et al., 2020). To account for variable read and gene lengths, gene counts analyzed from MetaStorm were normalized to 16S rRNA gene copy number to report relative abundance using Greengenes (DeSantis et al., 2006; Li et al., 2015). A list of clinically-relevant ARGs, defined as genes that confer resistance to key clinically-important antibiotics (e.g., peptides, glycopeptides, beta-lactams, and macrolides), were further analyzed (Keenum et al., 2021).

### ARG-MGE Co-occurrence and Resistome Risk Analysis

*De novo* assembled scaffolds were generated and annotated using the MetaStorm assembly pipeline for downstream ARG-MGE co-occurrence analyses (Arango-Argoty et al., 2016). In brief, MetaStorm uses IDBA-UD (Peng et al., 2012) to assemble scaffolds and employs PRODIGAL (Hyatt et al., 2010) to predict genes within each scaffold. Downstream taxonomic and functional gene annotation details are described in-depth in Arango-Argoty et al. (2016). Assembled scaffolds averaged 1,102, 504, and 1,066 bp for amendments, soils, and lettuce surfaces, respectively. Coverage of assembly varied among each sample type, from 0.2–74.5% of the total number of contigs. The average coverage of assembly of amendment and lettuce samples was 36.8 and 41%, respectively. This was much higher than the average assembly of soil samples, which was only 0.64% (Wind et al., 2020). Estimated average coverage of the amendment, soil, and lettuce samples were 40, 20.62 and 63%, as determined *via* Non-pareil (Rodriguez-R and Konstantinidis, 2014). Due to low assembly of the soil samples, which is to be expected giving its high complexity (Howe et al., 2014; Myrold et al., 2014; Chen et al., 2019); the ARG-MGE co-occurrences along each point of the pre-harvest vegetable production are most comparable within each sample type (i.e., amendment, soil, vegetable surface). Using MetaCompare, an emerging metagenomic risk computational tool (Oh et al., 2018), ARG-MGE co-occurrences were analyzed from assembled contigs and hypothetical resistome risk scores were calculated for each metagenome. The risk score, Q, was calculated by evaluating the occurrence of a scaffold that contained one or more co-occurring ARG, MGE, or pathogen gene marker (Oh et al., 2018). To reduce influence of potentially incorrectly assembled contigs, a threshold was set in which only ARG-MGE contig that were identified in two or more sequence replicates per sample type (i.e., amendment, soil, and vegetable surface) were included. This threshold was not achieved for any soil samples and they were therefore excluded from further assembly-based analysis.

### Taxonomic Analysis

Taxonomy was annotated *via* Kraken2 (Wood and Salzberg, 2014). Once taxonomy was assigned, genera abundance was determined *via* Bracken [Bayesian Re-estimation of Abundance with KrakEN, (Lu and Salzberg, 2020)]. Genera relative abundance files produced *via* Bracken were analyzed for microbial communities using R “vegan” package (Oksanen et al., 2015).

### Statistical Analysis

All statistical analyses were conducted using R (v. 4.0.3, R Core Team, 2020) with the alpha threshold of 0.05 (*p* < *0.05*) applied as the level of significance. All metagenomic datasets were confirmed as non-parametric *via* the Shapiro-Wilkes normality test (*p* < *0.05*). ARG relative abundance varied based on gene amplified and a Kruskal-Wallis rank sum test followed by a *post hoc* Dunn’s test assessed differences between time points, treatments, and among the amendment, soil, and lettuce surface metagenomes. On the assumption that prior to amendment application the field plots represented the same condition, the background (−1 day) soil metagenomic samples from the inorganic chemical fertilizer control, compost with antibiotics, and stockpiled manure with antibiotics amended soils were grouped with the no amendment control amended soil samples at the time of background, which resulted in six replicate samples during the statistical analysis (Supplementary Table 1). Bray-Curtis dissimilarity and a one-way analysis of similarities (ANOSIM) statistical analysis was used to compare resistome profiles (ARG type and ARG abundance).

The microbial community alpha diversity was measured using Shannon diversity index, which takes species proportional abundances into account for species richness. A one-way analysis of variance (ANOVA) followed by a *post hoc* Tukey HSD test assess differences among the amendment, soil, and lettuce surface microbial communities and subsequent treatment and time effects if needed at each point. Indicator species analysis of the significantly differentially abundant species was measured using point biserial correlation coefficient from the R package “indicspecies” (Cáceres, 2013). Figures were produced using R packages “ggplot2,” “VennDiagram,” and “networkD3” (Wickham, 2009; Gandrud et al., 2017; Chen, 2018).

## Results

### ARG Profiles

#### Comparison of Resistomes Across Sample Types

**FIGURE 1:**
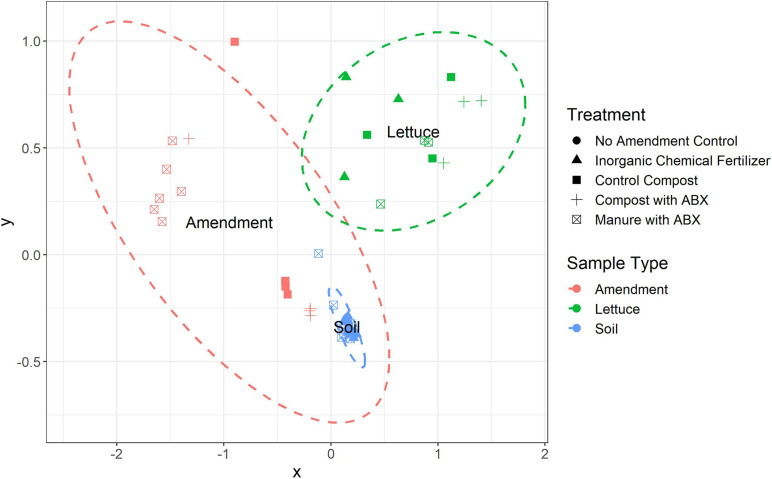
Non-metric multidimensional (NMDS) scaling of the Bray Curtis dissimilarity distances highlight the distinct ARG profiles among amendment, soil, and vegetable samples representing the pre-harvest vegetable production chain (ANOSIM, *R* = 0.85, *p* < 0.001). ARGs were annotated against the CARD v2.0.1 database.

**FIGURE 2:**
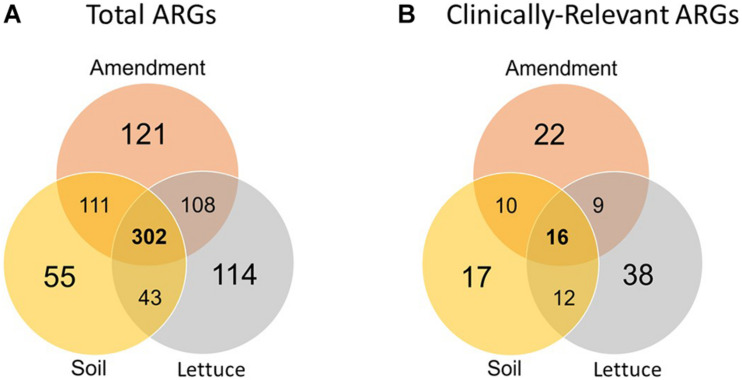
Number of unique **(A)** total and **(B)** clinically-relevant ARGs annotated *via* the CARD v2.0.1 database in the amendment, soil, and lettuce surfaces samples analyzed across the pre-harvest vegetable production chain.

The number of unique ARGs and their relative abundances differed significantly among the three sample types (i.e., amendment, soil, and lettuce; ANOSIM, *R* = 0.85, *p* < *0.0001*; Figure 1) across the pre-harvest vegetable production chain. Of the 854 unique ARGs annotated across all metagenomes, 302 were detected in all three sample types (Figure 2A) and a total of 121, 55, and 114 ARGs were unique to the amendment, soil, and lettuce samples, respectively (Shannon diversity, *p* < *0.0001*). Most importantly, 123 of the total ARGs were found to be clinically relevant to pathogens of concern for humans (Figure 2B). The greatest number of clinically-relevant ARG types were annotated in samples collected from the lettuce surfaces, with 16 clinically-relevant ARGs common among all amendment, soil, and lettuce samples. The lettuce grown in compost with antibiotics carried the highest clinically-relevant ARG relative abundance, while the lettuce grown in inorganic fertilizer had the least. The clinically-relevant ARGs with the greatest relative abundance overall were *bla*CARB-14, *bla*OXA-322, and *van*A. ARG Shannon diversity was significantly different when comparing amendment and lettuce samples (*p* < *0.001*) and the soil and lettuce samples (*p* < *0.0001*); but there was no significant difference between the amendment and soil samples (*p* = *0.07*).

#### Comparison of Amendment Resistomes Over Time

Resistome profiles of samples collected from stockpiled manure were distinct from those of the two composts (ANOSIM, *R* = 0.595, *p* < *0.001*; Supplementary Figure 2). Initial resistome profiles (0 day) of the compost pile and stockpiled manure also were different compared to those at the end (63 days; ANOSIM, *R* = 0.462; *p* = *0.011*; Supplementary Figure 2). The total ARG relative abundances were greatest in the compost with antibiotic, relative to the control compost or stockpiled manure (Kruskal-Wallis, *p* < *0.001*; *post hoc* Dunn Test, *p* < *0.001*) at both 0 day and 63 days (Kruskal-Wallis, *p* < *0.001*). The control compost and stockpiled manure with antibiotics were indistinguishable in terms of total ARG relative abundances (*post hoc* Dunn test, *p* = *0.63*; Supplementary Figure 3).

The ARG classes with the greatest relative abundance among the amendments were multidrug, tetracycline, macrolide-lincosamide-streptogramin (MLS), and glycopeptide (Supplementary Figure 3). The three most abundant ARGs were identical between the compost with and without antibiotics at 63 days (*par*Y, *mtr*A, and *van*RO) (Supplementary Figure 4), although the most abundant ARGs in the stockpiled raw manure were *tet*W, *Inu*A, and *efr*B (Supplementary Figure 4). Overall, 642 ARGs were annotated across the amendment samples and almost half, 316 ARGs, were common among all samples (Supplementary Figure 5). The control compost contained the most unique ARGs (*n* = 555), followed by the compost with antibiotics (*n* = 475) and the stockpiled manure (*n* = 405). The control compost also had the most ARGs distinct to a given sample type (*n* = 88), followed by the compost and stockpiled manure with antibiotics, with 43 and 34 distinct ARGs, respectively.

#### Comparison of Soil Resistomes With Amendment Type and Time

There were distinct differences in resistomes between the no amendment control soils and stockpiled manure-amended soils immediately following amendment (0 days); however, neither inorganic chemical fertilizer or composts amended soils measurably altered the respective soil resistome profiles relative to the control soils. By the time of lettuce harvest (67 days), all soil resistomes were similar (Wind et al., 2020), regardless of initial amendment condition. The total ARG relative abundance in all the soils across all amendment types were much greater than what was measured in the corresponding amendments, indicating that there were substantial naturally-occurring ARGs in the soil before the amendments were applied.

#### Comparison of Resistomes in Lettuce Grown With Different Amendments

The total ARG relative abundance in samples collected from the surface of lettuce grown in soils amended with compost or raw manure was twice that of the total ARG relative abundances recovered from the surfaces of the lettuce grown in soils amended with inorganic chemical fertilizer (Fogler et al., 2019). In all cases it is worth noting that the total ARG relative abundance levels recovered from the lettuce surfaces were up to 50% greater than in the amended soils in which they were grown. For example, lettuce grown in soil amended with stockpiled manure carried an average of 2 ARGs/16S rRNA gene, while the corresponding soil contained an average of 1.3 ARGs/16S rRNA gene, immediately after mixing with the amendments.

### Co-occurrence of ARGs

**FIGURE 3:**
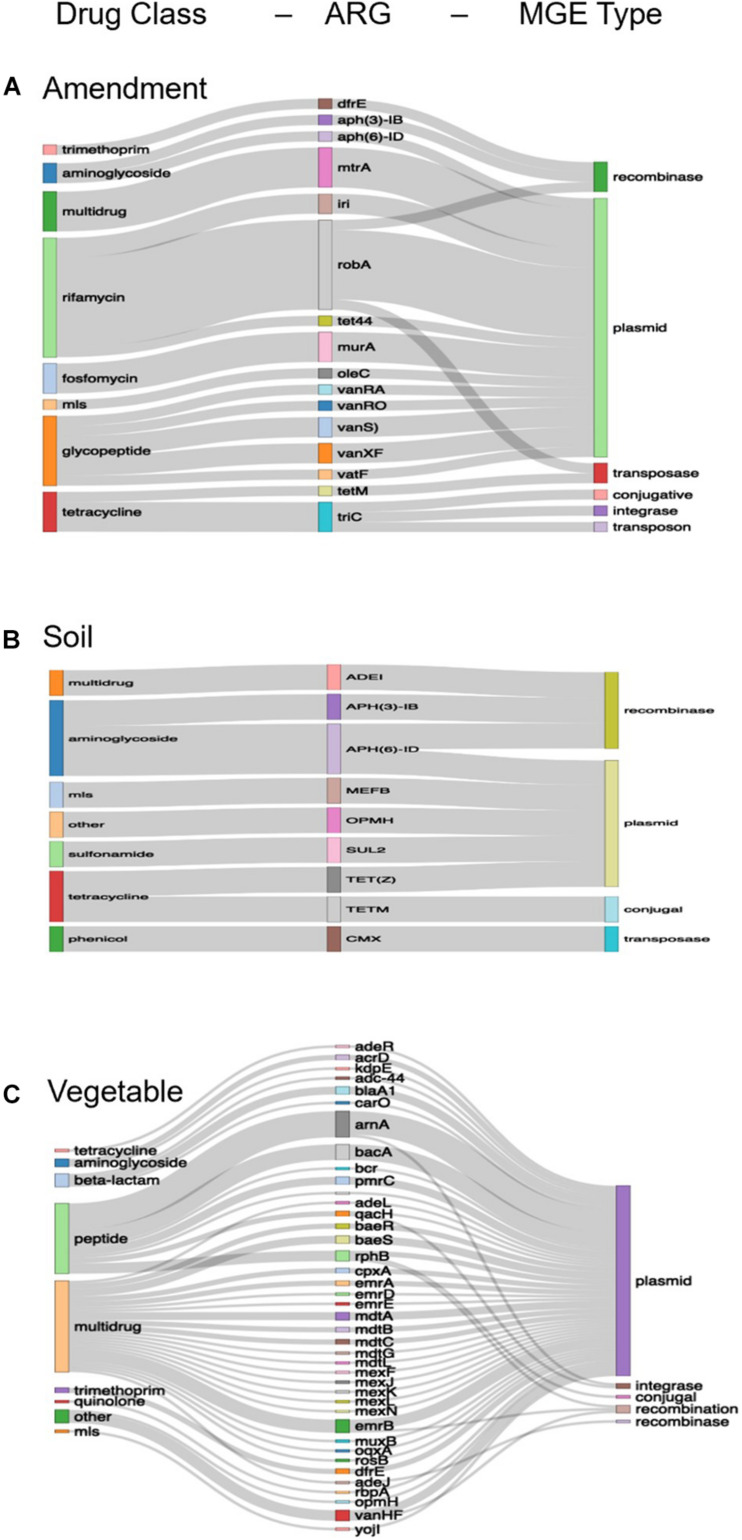
Unique ARG-MGE co-occurrences annotated among assembled contigs of the **(A)** amendment (*n* = 460), **(B)** soil (*n* = 22), and **(C)** lettuce (*n* = 1,221) categorized by drug resistance class, ARG type, and MGE type. The relative height of the bars for each category is proportional to the number of times the category was annotated in the samples and the thickness of the connecting ribbons corresponds to the frequency of the combination. For visualization purposes, the amendments and lettuce represent 460 and 1,221 unique ARG-MGE co-occurrences, respectively, meeting the threshold of having been annotated on at least two contigs within the sample type. However, the soil **(B)** represents all 22 unique ARG-MGE co-occurrences (without applying the threshold of 2 + common ARG-MGE contigs as there were none).

Following assembly of metagenomic data, there were 734, 22, and 4,761 unique ARG-MGE co-occurrences among the amendment, soil, and lettuce samples, respectively. After applying the threshold of ARG-MGEs contigs occurring at least twice in a given data set (i.e., across metagenomes from the same sample type), there were 460, 2, and 1,221 unique ARG-MGE co-occurrences among the assembled contigs from amendment, soil, and lettuce samples, respectively (Figures 3A–C).

Of the 460 unique ARG-MGE co-occurrences assembled within the amendment metagenomes, the most frequent one was between the rifamycin drug class and plasmids. The ARG *rob*A was the most prevalent on contigs that were also annotated with plasmid markers (Figure 3A). Due to low assembly for the soil samples, only 2 unique ARG-MGE co-occurrences were detected, both in the stockpiled manure amended soils (Figure 3B). The most frequent ARG-MGE co-occurrences in soil was between the aminoglycoside drug class and plasmids. The ARG *APH*(6)-id, which encodes resistance through antibiotic inactivation, was the most prevalent on plasmids annotated in the soils. Of the 1,221 unique ARG-MGE co-occurrences assembled on the lettuce surfaces, the most frequent co-occurrence was between the multidrug class and plasmids. The ARGs *am*A, *bac*A, and *erm*B were the most frequently co-occurring on the plasmids annotated on the lettuce surfaces.

### Relative Resistome Risk Comparison

Among the analyzed samples, all amendment and lettuce samples contained scaffolds that were annotated with co-occurring ARGs, MGEs, and human pathogen markers, which increased their resistome risk scores. Overall, the resistome risk scores were significantly different among the three sample types (Kruskal-Wallis, *p* < *0.001*; *post hoc* Dunn, *p* < 0.001 for all iterations; Supplementary Figure 6). The average resistome risk scores were 26.9, 22.6, and 60.1 for the amendments, soils, and lettuce, respectively (Supplementary Table 2). On average, there were 375, 13, and 1,420 contigs annotated with pathogen gene markers among the three sample types, respectively. Within each sample type, the resistome risk scores were comparable, regardless of associated experimental conditions or time points (Kruskal-Wallis, *p* > *0.05* for all iterations; Supplementary Figure 7).

### Taxonomic Composition Comparison

**FIGURE 4:**
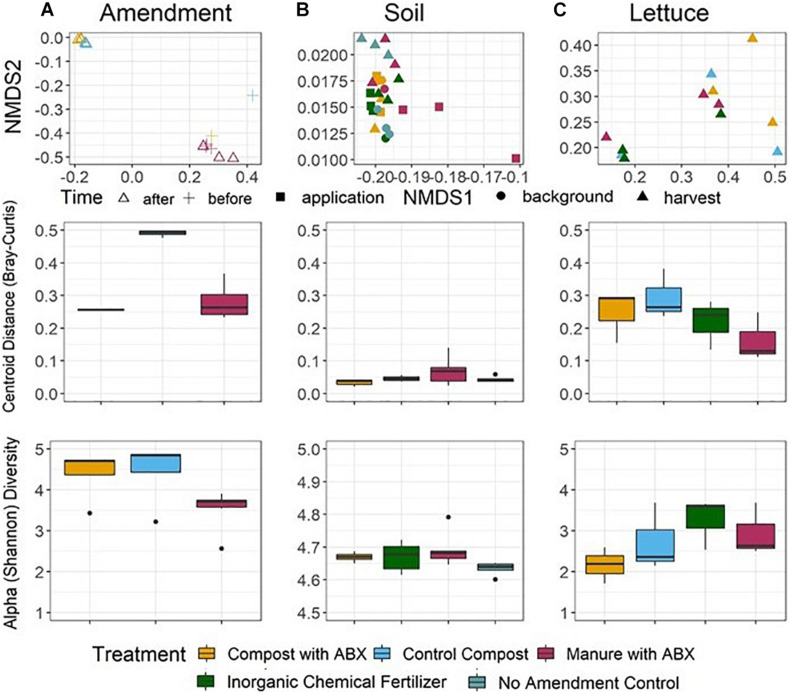
Taxonomic alpha (bottom row) and beta (top and middle rows) diversity across the microbial community’s pre-harvest vegetable production chain for the **(A)** amendment, **(B)** soil, and **(C)** lettuce. Alpha diversity was calculated as Shannon diversity. Beta Diversity was determined from Bray-Curtis dissimilarity matrices and visualized as NMDS (top panel) and distance to euclidean centroid (middle panel). Taxonomy was annotated using NBCI taxonomy id using Kraken2 as the metagenomic classification algorithm and Bracken2 to compute species abundance.

Microbial communities recovered from the amendment, soil, and lettuce surface samples were distinct in terms of taxonomic composition (ANOVA, *p* < *0.001*; ANOSIM, *R* = 0.156, *p* = *0.0104*; Figure 4, Supplementary Figure 8) and Shannon diversity (*post hoc* Tukey HSD, *p* < *0.001* for all iterations) across the pre-harvest vegetable production chain. Both composts and raw manure amendments were also distinct from each other in terms of Shannon diversity (ANOVA, *p* = *0.0114*), but there was not a significant alpha diversity difference in taxonomic composition among the soil samples or among the lettuce samples in terms of the amendment conditions (ANOVA, *p* = *0.0687* and *0.287*, respectively) or at different time points (ANOVA, *p* = *0.795*).

Bray-Curtis dissimilarity matrixes also indicated differences in beta diversity of taxonomic profiles among the amendments, soils, and lettuce surface samples. Further, differences could be discerned among the amendment samples and soil samples according to corresponding experimental conditions (ANOSIM, *p* < *0.001* for both). Contrary to the resistome analysis, there was no Bray-Curtis dissimilarity difference in taxonomic profiles between the microbial communities before (0 day) versus after (63 days) amendment treatment (ANOSIM, *p* = *0.613*) or in the soils over the growing season (−1, 0, and 67 days) (ANOSIM, *p* = *0.147*).

Among all the agricultural metagenomes, 567 unique genera were identified, with 25% uniquely found in each sample type. Indicator species analysis *via* the point biserial correlation coefficient highlighted the significant differentially abundant genera at and between the amendments, soils, and lettuce surfaces samples (*p* < 0.01 for all iterations, Supplementary Table 4). Amendments had the most diverse set of differentially abundant genera, including 197 unique genera not found in other sample types. Soil and lettuce samples contained 129 and 31 genera that were differentially abundant, respectively. The top indicator genera (i.e., bacteria whose abundances were significantly different between the sample types) for the amendments were *Parageobacillus, Aneurinibacillus*, and *Belliella* (*p* < 0.0001 for all). The top indicator genera of the amended soils were *Bradyrhizobium, Mycobacterium*, and *Rhodoplanes (p* < *0.0001* for all). The top indicator genera of the lettuce surfaces were *Pseudomonas, Pantoea*, and *Massilia* (*p* < *0.0001* for all). *Mycobacterium* identified in the soil and the *Pseudomonas* and *Pantoea* identified on the lettuce are considered to be opportunistic pathogens with respect to human health. On average, 15 and 12% of the classified bacteria may be considered pathogenic among the amendment and soil samples, respectively. The most abundant potential pathogenic genera included *Pseudomonas* in the composts and *Clostridium* and *Enterococcus* in the manure amendments, and *Pseudomonas* and *Mycobacterium* in the soils. On average, 36.7% of the classified bacteria may be considered potentially pathogenic among the lettuce surface samples. The most abundant potential pathogenic genera, up to 90% on the majority of lettuce samples, included *Pseudomonas*.

## Discussion

This integrated study provided a comprehensive assessment of various pre-harvest factors as critical control points shaping resistomes in vegetable production systems that is not possible from examining points in the system individually. The results highlight the importance of holistically considering multiple control points at which antibiotic resistance might spread, or be controlled, through a vegetable production system. Such points should be considered further as candidates for targeted mitigation strategies; including the use of antibiotics, composting manure prior to land application, and amendment of soils with organic fertilizers, which were all observed to influence the lettuce resistomes in some manner. We found that where effects of these factors were readily measurable in the resistomes of the organic amendments and the harvested lettuce, they were only transiently measurable within the soil following amendment. Remarkably, total ARG relative abundance increased from amendment to soil to lettuce surface across all conditions, except in the inorganic chemical fertilizer condition. The results highlight that, even if attenuation of resistance markers appears to be occurring during manure management and after soil application, that there still could be lingering concerns regarding the potential for ARBs and ARGs to be selectively transferred and enriched on the crop surface.

### Effects of Manure Collection During Antibiotic Administration

Freshly collected manure (0 days) from the cows administered pirlimycin or cephapirin contained a higher total ARG relative abundance than manure collected from controls cows. Further, antibiotic administration had a lingering effect on the resistome after composting or stockpiling the manure for 63 days (Supplementary Figures 3, 4). Stockpiled manure from cows treated with antibiotics contained substantially more ARG-MGE co-occurrences compared to the compost derived from control cows, suggesting that lack of antibiotic use and composting together reduced ARG mobility. Within the stockpiled manure, the *TET*M to transposon (*Clostridium difficile*) co-occurred 12 times and was the most abundant ARG-MGE annotated across the pre-harvest vegetable production chain. However, the overall resistome risk score of the control compost was higher than both the stockpiled manure and antibiotic compost. The reasons for this are unclear, but could relate to the higher taxonomic beta diversity observed in the control compost (Figure 3A) supporting a greater variety of ARG-MGE and potential ARG-MGE-pathogen contigs which aligns with the taxonomic profiles indicating higher beta diversity of the control compost. This finding is counter to the expectation that antibiotic use elevates the risk of spreading resistance.

### Effects of Composting

The average total ARG relative abundances were elevated in composted manure as compared to stockpiled manure after 63 days of manure management (i.e., composting). This agrees with previous studies that have observed an increase in ARG abundance following composting (Storteboom et al., 2007; Xu et al., 2019; Keenum et al., 2021). Although composting is recommended to mitigate pathogens (FDA, 2015), the benefits might not extend to ARG attenuation. Interestingly, the control compost was the most diverse in terms of number of ARGs annotated (Supplementary Figure 5) among the three amendments, suggesting that the antibiotics had a lingering effect of suppressing taxonomic diversity (Figure 4 and Supplementary Figure 8), which carried over into the observed diversity of the resistome. However, the higher diversity of ARGs in the control compost did not translate into higher diversity of ARGs detected in the amended soil or on the cultivated lettuce.

The most prominent classes of antibiotics that the ARGs corresponded to also differed based on manure management practice. By 63 days, the compost had higher relative abundances of glycopeptide ARGs as compared to the stockpiled manure. On the other hand, the stockpiled manure contained higher levels of MLS and tetracycline ARGs than were annotated in the composts (Supplementary Figure 3). The differences in ARG profiles categorized by antibiotic class at the end of manure treatment indicate that composting was able to mitigate bacteria conferring resistance to antibiotics that are clinically-important for human health (WHO, 2017). However, there was no significant difference in taxonomic profiles between the stockpiled manure and compost at 63 days, which suggests that the resistome is not merely a function of the taxonomic composition of the microbial community and that there are distinct ecological drivers (e.g., horizontal gene transfer) shaping ARG profiles during composting (Qian et al., 2016; Zhou and Yao, 2020).

### Effects of Amending Soil

There were significant resistome (diversity and abundance) differences between the soil amended with stockpiled manure containing antibiotics and the no amendment control soil resistomes; but there was no difference among the total ARG relative abundances quantified between all the amended soils with respect to time (Wind et al., 2020). From this finding, we hypothesize that one growing season was not enough time to predict the long-term impact of potential amendment addition of ARGs in agricultural soils. Recent organically fertilized agricultural soil studies have identified similar patterns of ARG levels and resistomes retuning to background after a single growing season (Zhou et al., 2017; Macedo et al., 2021). Multiple years of application may be required to appreciably alter soil resistomes (Liu et al., 2021).

From a clinical perspective, the soil contained the least ARG-MGE co-occurrences compared to the other points of the pre-harvest vegetable production chain (22 soil ARG-MGE compared to the 734 and 4,761 annotated within the amendment and lettuce surface samples, respectively) and the soil was characterized by significantly lower resistome risk scores (Supplementary Figures 6, 7). However, it is important to consider that the lower assembly rate achieved due to the soil complexity likely influenced the resistome risk score. Still, it should be pointed out that the only ARG-MGE co-occurrences that were found in soil were in the stockpiled manure amended condition. This suggests that the stockpiled manure amended soils may ultimately contain more ARG-MGE co-occurrence than the other soils if deeper sequencing is utilized in the future to explore the amended soils.

### Lettuce Surface Resistomes

The total ARG abundance recovered from lettuce grown in manure- or compost-amended soil was 2−3 × greater than that collected from lettuce grown in soils amended with inorganic fertilizer. This demonstrated a clear effect of organic soil amendments on lettuce resistomes. Notably, even though measurable differences in soil resistomes associated with organic amendments were lost in soil, they re-emerged on the lettuce. Further, although composting had resulted initially in a larger diversity of ARGs compared to the manure (Supplementary Figures 3, 5), there did appear to be benefits in terms of the lettuce grown in control compost-amended soils carrying the lowest ARG relative abundance among the organically-amended soil conditions (Fogler et al., 2019). It is important to highlight that although manure management had an effect on the control compost in terms of ARG abundances compared to the stockpiled manure, no effect was seen when comparing the lettuce grown in the soils amended with control compost to lettuce grown in the soils amended with compost derived from antibiotic-administered cow manure. Lettuce grown in soils amended with compost generated from the manure of treated cows had higher total ARG relative abundance and risk scores compared to the stockpiled manure. In a recent study, Zhang Y. J et al. (2019) similarly found that manure (poultry) application to the soil increased ARG abundance on lettuce surfaces at the greenhouse scale.

Remarkably, when comparing the three sample types (organic amendment, soil, lettuce), there were several concerning features of lettuce resistomes that were identified. For example, the highest abundance of clinically-relevant ARGs (*n* = 75) and the most ARG-MGE co-occurrences were found in lettuce samples. The *Tri*C to plasmid (*Ralstonia solanacearum*) co-occurred 10 times on the lettuce surfaces grown in the compost and was the second most abundant ARG-MGE. The higher number of ARG-MGE co-occurrences and, at times, higher pathogen detection on the lettuce surface contigs (Supplementary Table 3) is concerning from a human health perspective and is an area needing further research due to the potential for horizontal gene transfer (Zhang Y. J et al., 2019). Recent studies have focused specifically on vegetable surface resistomes and have found that post-harvest sanitization practices, including irradiation and washing in water containing sanitizers, reduces surface ARGs (Dharmarha et al., 2019).

### Pre-harvest Conditions on Taxonomic Composition

Taxonomic compositions were distinct among each of the three sample types (Figure 4). The most common genera detected in the organic amendments belonged to the *Firmicutes* and *Bacteroides* phyla, which is to be expected, given that these are associated with cattle gut microbiomes and fecal bacteria. The top soil genera belonged to the *Proteobacteria* and *Actinobacteria* phyla, which are known to contain pathogenic bacteria relevant to agricultural and terrestrial systems. The top vegetable genera also corresponded to the *Proteobacteria* phyla. Although the vegetables surfaces carried the least indicator genera (*n* = 31), it should be noted that most of the ones highlighted here are classified as opportunistic pathogens with environmental niches [e.g., *Pseudomonas* (skin, ear, and lung infections), *Panteo* (septic arthritis), and *Massilia*, which poses risk of enteric infections]. The amendment and soil microbial communities were the most similar, containing 124 indicator genera that were significantly associated between the two points (*p* < 0.001). The top three genera shared between amendments and soils included *Spirochete*, *Acetobacter*, and *Leptospirillum.* Soil and vegetable microbial communities were the least similar to each other, containing only five indicator genera that were significantly associated between the two points (*p* < 0.001). The top three genera between the soil and vegetable surfaces included *Nocardioides, Methylorubrum*, and *Pseudarthrobacter*. The common genera between the pre-harvest vegetable production chain points may be indicative of a transfer of specific microbial taxa, including pathogens, within agroecosystems. Surprisingly, lettuce surface samples had on average almost 3 × and 2.5 × greater percent of the classified bacterial abundance that could be considered pathogenic than the soil and amendments, respectively (Supplementary Text 1 for list of pathogens). Further microbial community analyses are needed to connect microbial community diversity and ARG attenuation or proliferation, and deeper taxonomic analysis than the genera level may provide insight to potential pathogenicity which will allow for bacterial host identification across the pre-harvest vegetable production chain. Other studies have indicated need to examine this more closely specifically in agricultural soils (Cycoń et al., 2019; Lopatto et al., 2019) and on vegetable surfaces (Zhang Y. J et al., 2019; Shen et al., 2021).

### Limitations

To our knowledge, this study is the first of its kind to bridge the pre-harvest vegetable production chain resistomes through a controlled field-scale study applying metagenomics. However, it is critical to explicitly examine analytical and experimental limitations. With any next-generation sequencing approach, there are inherent database bias, sampling processing bias, and varying annotation thresholds to acknowledge. First, due to the archival nature of this study and changing sequencing platforms and methodologies with time, shotgun metagenomic sequencing was carried out on different Illumina instruments for the amendment and soil versus lettuce samples. While we were able to account for such differences in our analysis and interpretation, in the ideal situation, one would conduct such studies in the future with a uniform protocol. However, that will not always be possible, especially as more and more studies seek to harness data analytics toward harvesting the growing volumes of valuable sequencing information available in public databases.

Given the biological richness and physiochemical complexity of the soil ecosystem, it is perhaps not surprising that the soil samples had the lowest percent assemblies of the shotgun metagenomic data, and subsequently contained the least annotated ARGs (*n* = 511) compared to the amendments (*n* = 642) and the lettuce surfaces (*n* = 567). Low sequencing coverage from shotgun metagenomic data in soils is a widely recognized issue (Myrold et al., 2014; Zaheer et al., 2018). The low sequence coverage and percent assembled contigs from soil metagenomes may have resulted in missing the key ARG-MGE co-occurrences at this specific pre-harvest vegetable production chain point. The low percentage of assembled contigs recovered from soil not only produced two to threefold less ARG-MGE co-occurrences, but also could have been a contributing factor to the low resistome risk score calculated using assembled contigs *via* MetaCompare (Oh et al., 2018). In theory, normalization to total recovered contigs in the determination of the resistome risk score should account for such differences, but this may not be the case for situations of extremely low assembly rates, which may not be representative and thus skew the calculation.

There was some variation in sequencing depth across the sample types, with vegetable samples (18 million reads) averaging ∼80% of reads obtained for amendment and soil (∼23 million reads, Supplementary Table 2). Still, there was almost twofold and threefold higher relative abundance of ARGs detected on the lettuce surfaces and amendments, respectively, than in soil. If sequencing coverage would have been a factor, then the opposite trend would have been anticipated. These trends are consistent for soil as soil microbial communities are inherently more diverse than any other environmental microbial community, and therefore the low coverage obtained is anticipated (Myrold et al., 2014; Zaheer et al., 2018). Additionally, the increase in assembled contigs subsequently produced more ARG-MGE co-occurrences and subsequently increased resistome risk estimates for lettuce, compared to the amendment and soil samples. A possible explanation of the increase in assembly is due to the higher sequencing coverage (∼40%) found for the lettuce samples. It is important to mention that the lettuce surface sequences had chloroplast sequences removed prior to annotation (Fogler et al., 2019). Although it is not surprising that the resistome characterized from each environmental sample type (amendment, soil, lettuce surface) was different, the magnitude of these differences was surprising, and suggests a need for future efforts to examine these inconsistencies at a more robust biological level. Overall the differences in sequencing platforms, coverage and assembly across the pre-harvest vegetable production chain points should be acknowledged and accounted for in future when comparing critical control points and environmental studies (Liang et al., 2021).

## Conclusion and Recommendations

Agroecosystems are a key reservoir of antibiotic resistance and appropriate mitigation strategies are needed to limit potential for ARGs to spread and negatively influence human and animal health. This study employed metagenomic analysis to provide an integrated assessment of amendment, soil, and lettuce resistomes and highlighted the importance of characterizing multiple hypothetical control points that shape resistome at field-scale, including antibiotic use, composting manure prior to soil amendment, soil composition, and cultivation of lettuce. The findings from the current study emphasize the importance of multiple agricultural best management practices to provide a multi-barrier approach for preventing the spread of ARGs. Antibiotic administration to animals and composting of manure were found to have measurable effects on the harvested lettuce resistomes. The soil was found to be highly stable in the composition of the resistome and corresponding resistomes and has the potential to act as a natural ecological buffer to ARG proliferation, at least following one harvest cycle. Notably, amendment application at standard agronomic rates did not alter soil resistomes or microbial community taxonomic composition after one growing season compared to background levels. Further studies are recommended to determine if there is variance among soil types in their capacity to buffer the potential for ARGs to spread.

Still, total ARG relative abundances were higher in the lettuce phyllosphere than in the soil or amendments and were highest on lettuce grown in soils receiving organic compared to inorganic amendments. This indicates that the transient impacts observed in soil only immediately after amendment had lasting impacts on the phyllosphere resistomes. Lettuce was characterized by the greatest number of ARG-MGE co-occurrences as well as resistome risk scores. Further research examining effects of post-harvest practices, such as washing, packaging, and storage on vegetable resistomes, are needed. Moving forward, it would be beneficial address the clinical ARGs and other forms of antibiotic resistance from a One Health lens (i.e., which clinical ARGs are we most concerned about for humans, animals, and the environment) and then shift detection methods to identifying the most clinically important co-occurring ARG-MGE that are associated with known pathogens to improve future agricultural antibiotic resistance mitigation strategies.

## Data Availability Statement

The manure obtained from the dairy cows was from an animal study reviewed and approved by IACUC protocol DASC 13-145.

## Ethics Statement

The animal study was reviewed and approved by IACUC protocol DASC 13–145.

## Author Contributions

LW: formal analysis, writing—original draft preparation. L-AK, AP, WH, LW, PR, KK, MP, and SG: conceptualization. LW, IK, SG, L-AK, AP, and WH: methodology, writing—review and editing. L-AK and AP: resources, funding acquisition, project administration, and supervision. L-AK, AP, WH, and IK: writing—revising and editing. All authors contributed to the article and approved the submitted version.

## Conflict of Interest

The authors declare that the research was conducted in the absence of any commercial or financial relationships that could be construed as a potential conflict of interest.
