# Supplementary material for: Integrated Metagenomic Assessment of Multiple Pre-harvest Control Points on Lettuce Resistomes at Field-Scale
Source: Front Microbiol. 2021 Jul 9;12:683410. doi: 10.3389/fmicb.2021.683410 (PMC8299786; doi:10.3389/fmicb.2021.683410)
Supplement: Supplementary file 1 [file Data_Sheet_1.docx]

**Supplementary Material**

Integrated Metagenomic Assessment of Multiple Pre-harvest Control Points on Lettuce Resistomes at Field-Scale

Lauren Wind^1^, Ishi Keenum^2^, Suraj Gupta^3^, Partha Ray^4,5^, Katharine Knowlton^4^, Monica Ponder^6^, W. Cully Hession^1^, Amy Pruden^2^, Leigh-Anne Krometis^1^

^1^ Department of Biological Systems Engineering, Virginia Tech, Blacksburg, VA, United States

^2^ Department of Civil and Environmental Engineering, Virginia Tech, Blacksburg, VA, United States

^3^ The Interdisciplinary PhD Program in Genetics, Bioinformatics, and Computational Biology, Virginia Tech, Blacksburg, VA, United States

^4^ Department of Dairy Science, Virginia Tech, Blacksburg, VA, United States

^5^ Department of Dairy Sciences, School of Agricultural, Policy and Development, University of Reading, Reading RG6 6EU, UK

^6^ Department of Food Science & Technology, Virginia Tech, Blacksburg, VA, United States

^*^ Corresponding Author: wlauren@vt.edu

Text S1. Pathogens included: "Bacillus", "Bartonella", "Bordetella", "Borrelia", "Brucella", "Campylobacter", "Chlamydia", "Clostridium", "Corynebacterium", "Enterococcus", "Escherichia", “Francisella", "Haemophilus", "Helicobacter", "Legionella", "Leptospira", "Listeria", "Mycobacterium", "Mycoplasma", "Neisseria", "Pseudomonas", "Rickettsia", "Salmonella", "Shigella", "Staphylococcus", "Streptococcus", "Treponema", "Ureaplasma", "Vibrio", "Yersinia" found on CDC and WHO known lists.

Table S1. Detailed replication schematic of the metagenomes.

| **Matrix** | **Treatment** | **Time** | **Replicates** | **n** |
| --- | --- | --- | --- | --- |
| **Amendment** | (1) Control Compost | Before (0d) | 1 | **14** |
|  |  | After (63) | 3 |  |
|  | (2) Compost with Antibiotics | Before (0d) | 1 |  |
|  |  | After (63) | 3 |  |
|  | (3) Stockpiled manure with Antibiotics | Before (0d) | 3 |  |
|  |  | After (63) | 3 |  |
| **Amended Soil** | (1) No Amendment Control | Background (-1d) | 3 | **27** |
|  |  | Harvest (67d) | 3 |  |
|  | (2) Inorganic Chemical Fertilizer | Background (-1d) | 1 |  |
|  |  | Application (0d) | 3 |  |
|  |  | Harvest (67d) | 3 |  |
|  | (3) Compost with Antibiotics | Background (-1d) | 1 |  |
|  |  | Application (0d) | 3 |  |
|  |  | Harvest (67d) | 3 |  |
|  | 4) Stockpiled manure with Antibiotics | Background (-1d) | 1 |  |
|  |  | Application (0d) | 3 |  |
|  |  | Harvest (67d) | 3 |  |
| **Lettuce Surface** | (1) Inorganic Chemical Fertilizer | Harvest (67d) | 3 | **12** |
|  | (2) Control Compost | Harvest (67d) | 3 |  |
|  | (3) Compost with Antibiotic | Harvest (67d) | 3 |  |
|  | (4) Stockpiled manure with Antibiotics | Harvest (67d) | 3 |  |

Table S2. Read Matching and Assembly Sequencing Metadata

| Table S3. Average Resistome Risk Scores at each point along the vegetable production chain determined via MetaCompare. | | | | | | | | | | |
| --- | --- | --- | --- | --- | --- | --- | --- | --- | --- | --- |
| **Sample Type** | **Treatment** | **Time** | Contigs | ARG | MGE | PAT | Q  (ARG) | Q  (ARG_MGE) | Q  (ARG_MGE_PAT) | Risk_Score |
| **Lettuce Surface** | Compost with Antibiotics | Harvest | 63564.0 | 510.0 | 1229.3 | 1767.0 | 0.009847 | 0.001772 | 0.000697 | 97.44 |
|  | Control Compost | Harvest | 78890.7 | 455.3 | 1194.3 | 1121.3 | 0.005770 | 0.001035 | 0.000376 | 52.65 |
|  | Inorganic Chem Fertilizer | Harvest | 44712.0 | 242.0 | 338.3 | 807.0 | 0.006084 | 0.000207 | 0.000037 | 37.59 |
|  | Manure with Antibiotics | Harvest | 93396.3 | 580.0 | 1276.7 | 1986.3 | 0.006238 | 0.000945 | 0.000266 | 52.73 |
| **Soil** | Compost with Antibiotics | Background | 15151.0 | 39.0 | 162.0 | 11.0 | 0.002574 | 0.000000 | 0.000000 | 24.17 |
|  | Inorganic Chem Fertilizer | Background | 5731.0 | 5.0 | 120.0 | 1.0 | 0.000872 | 0.000000 | 0.000000 | 19.56 |
|  | Manure with Antibiotics | Background | 4266.0 | 5.0 | 69.0 | 2.0 | 0.001172 | 0.000000 | 0.000000 | 20.30 |
|  | No Amendment Control | Background | 7396.3 | 9.0 | 96.7 | 2.3 | 0.001194 | 0.000000 | 0.000000 | 20.36 |
|  | Compost with Antibiotics | Time of Application | 5672.3 | 8.0 | 108.7 | 2.0 | 0.001310 | 0.000000 | 0.000000 | 20.71 |
|  | Inorganic Chem Fertilizer | Time of Application | 8145.0 | 11.7 | 169.0 | 1.3 | 0.001437 | 0.000000 | 0.000000 | 21.00 |
|  | Manure with Antibiotics | Time of Application | 14693.3 | 70.0 | 253.0 | 95.7 | 0.004441 | 0.000517 | 0.000129 | 37.00 |
|  | Compost with Antibiotics | Harvest | 6142.3 | 6.7 | 99.0 | 1.0 | 0.001060 | 0.000000 | 0.000000 | 20.02 |
|  | Inorganic Chem Fertilizer | Harvest | 8797.7 | 14.7 | 146.0 | 4.7 | 0.001562 | 0.000000 | 0.000000 | 21.37 |
|  | Manure with Antibiotics | Harvest | 5761.0 | 9.3 | 105.7 | 1.7 | 0.001668 | 0.000000 | 0.000000 | 21.63 |
|  | No Amendment Control | Harvest | 5169.3 | 6.3 | 105.3 | 0.7 | 0.001113 | 0.000000 | 0.000000 | 20.16 |
| **Amendment** | Compost with Antibiotics | Before | 110814.0 | 326.0 | 1477.0 | 467.0 | 0.002942 | 0.000334 | 0.000108 | 27.38 |
|  | Control Compost | Before | 128508.0 | 621.0 | 2123.0 | 959.0 | 0.004832 | 0.000521 | 0.000210 | 37.38 |
|  | Manure with Antibiotics | Before | 153790.5 | 365.0 | 1386.5 | 511.5 | 0.002374 | 0.000326 | 0.000120 | 25.41 |
|  | Compost with Antibiotics | After | 108475.3 | 291.7 | 1260.7 | 84.7 | 0.002690 | 0.000289 | 0.000021 | 25.87 |
|  | Control Compost | After | 102768.0 | 295.7 | 1123.3 | 168.3 | 0.002909 | 0.000240 | 0.000039 | 26.49 |
|  | Manure with Antibiotics | After | 173146.3 | 442.3 | 1638.0 | 554.3 | 0.002553 | 0.000296 | 0.000117 | 25.87 |


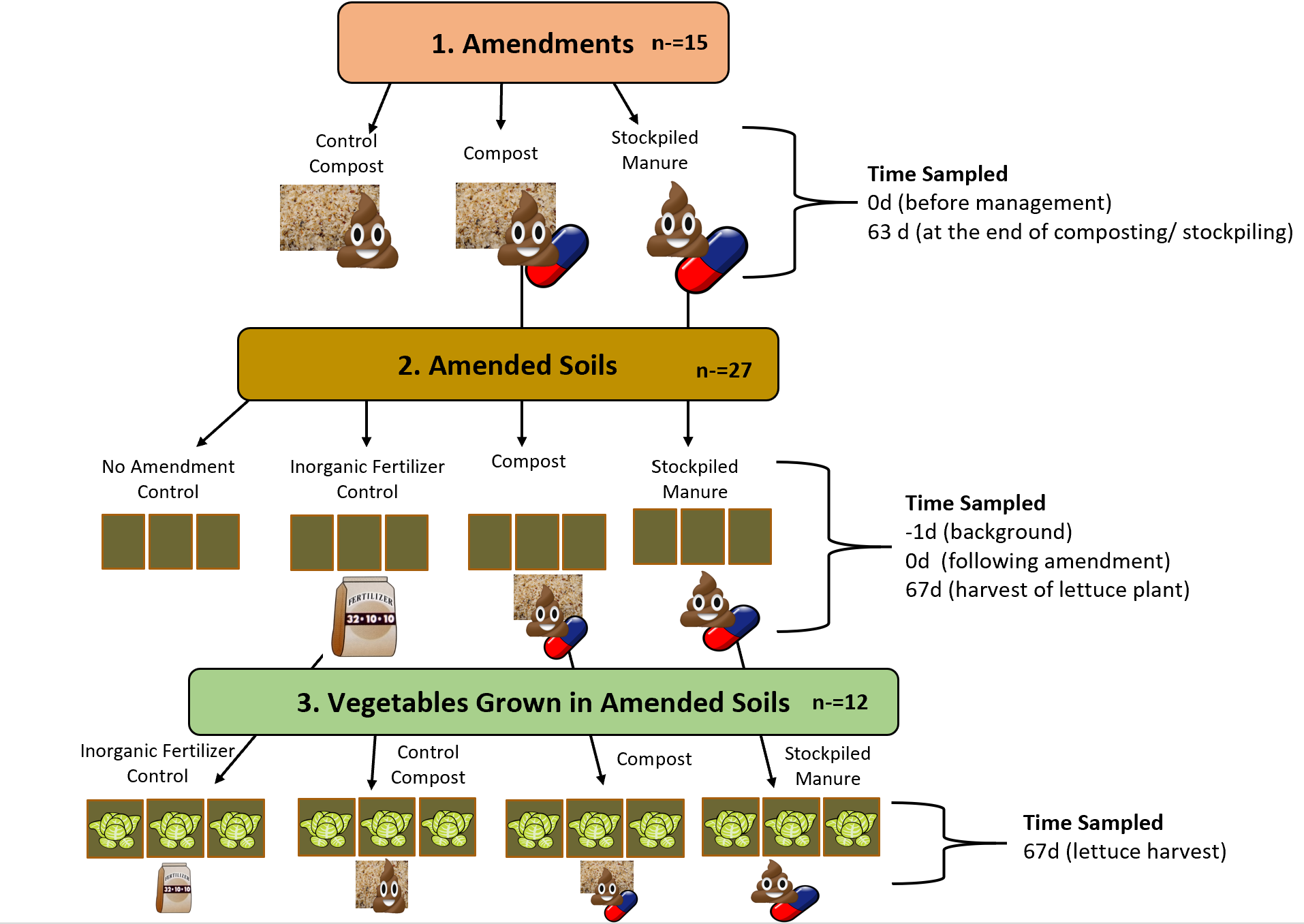


Figure S1. Study design flowchart of the field-scale pre-harvest vegetable production chain employed in this study. Point 1: Compost mixtures of stockpiled manure without antibiotics (control) and with antibiotics was examined before and after composting and compared to stockpiled manure with antibiotics before and after stockpiling during the same period. Point 2: four triplicate soil plots were amended with the compost with antibiotics and stockpiled manure with antibiotic amendments from point 1 and compared to an inorganic chemical fertilizer control and to a no amendment control. Point 3: Lettuce were grown in and harvested from the soil plots for the three amendments examined in Point 2 + plots receiving a fourth amendment of no antibiotic control compost. Surficial microbiota on the lettuce phylosphere were examined. Note: no lettuce was grown in the no amendment control soils because they were nutritionally-lacking.


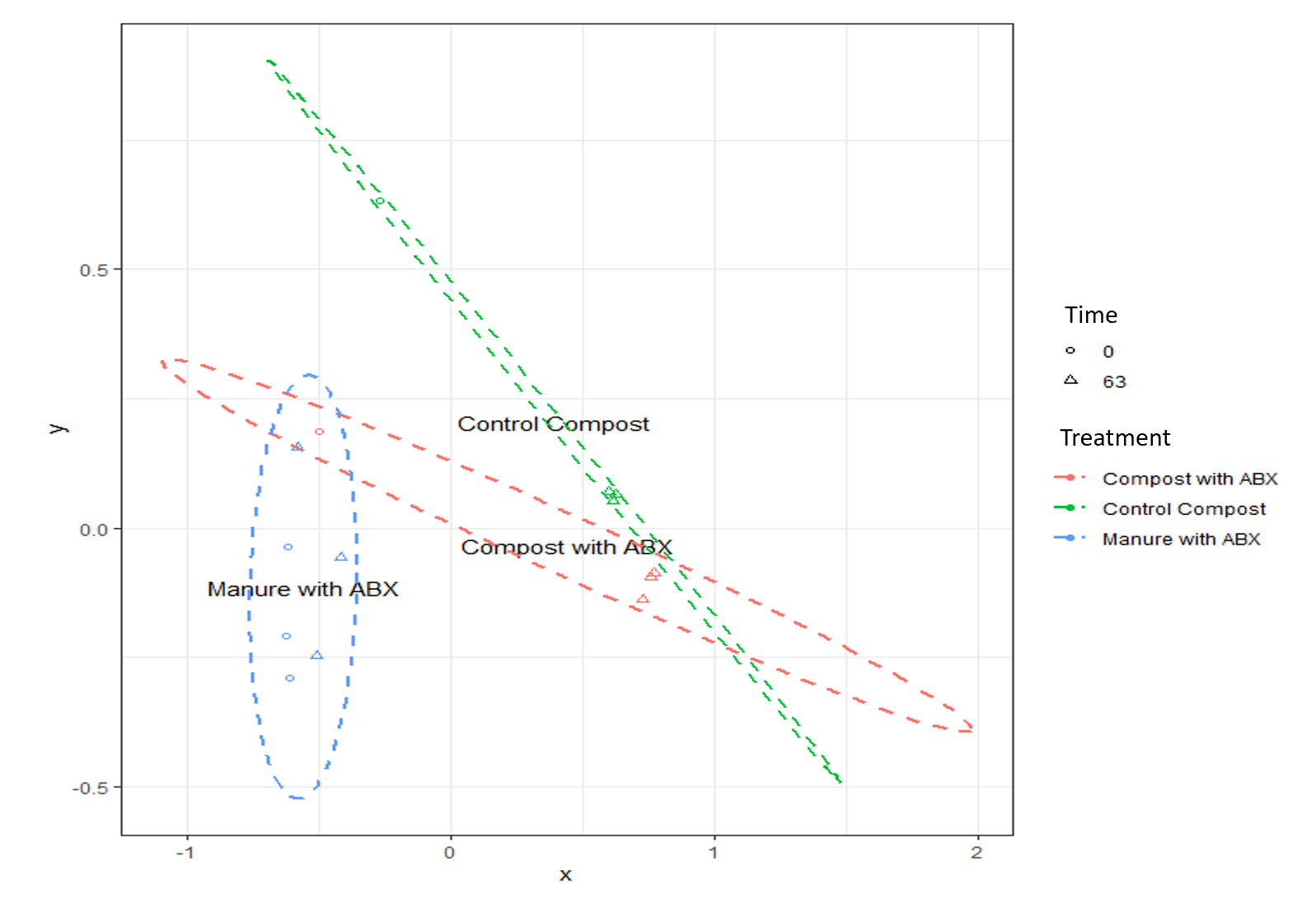


Figure S2. Non-metric multidimensional (NMDS) scaling of the Bray Curtis dissimilarity distances highlight the distinct ARG profiles among the amendment types, control compost, compost with antibiotics, and manure with antibiotics. (ANOSIM, R=0.595, p<0.001). ARGs were annotated against the CARD v2.0.1 database.


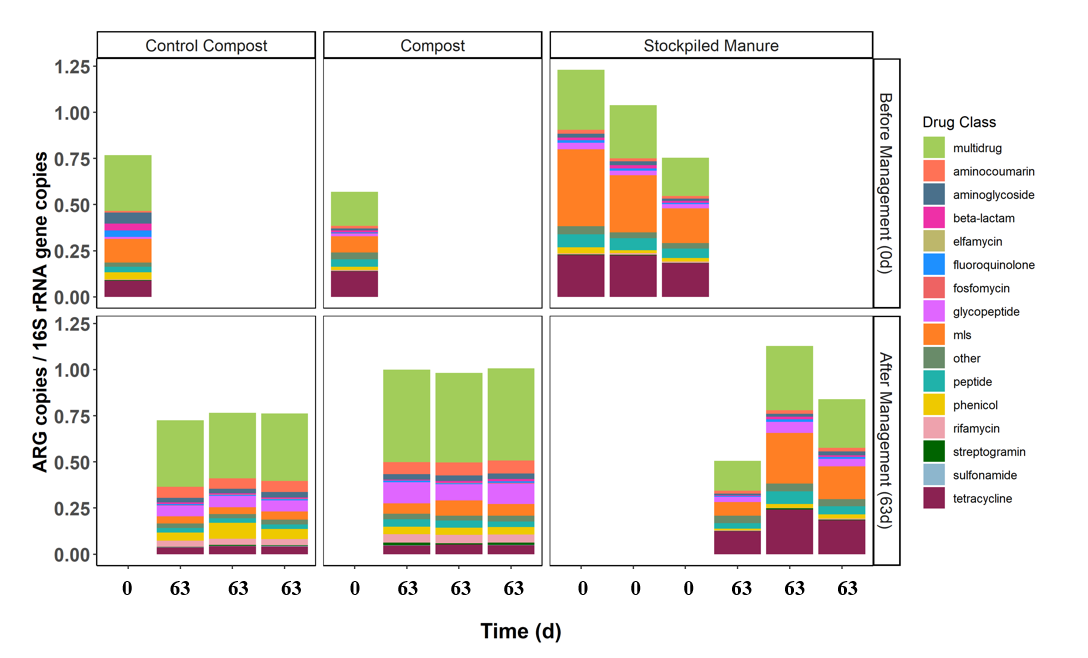


Figure S3. Amendment resistome profiles demonstrated as the relative abundance of ARGs before (0d) and after (63d) stockpiling and composting processes represented according to class of antibiotic to which they encode resistance. ARGs were annotated using CARD v2.0.1 via shotgun metagenomic sequencing.


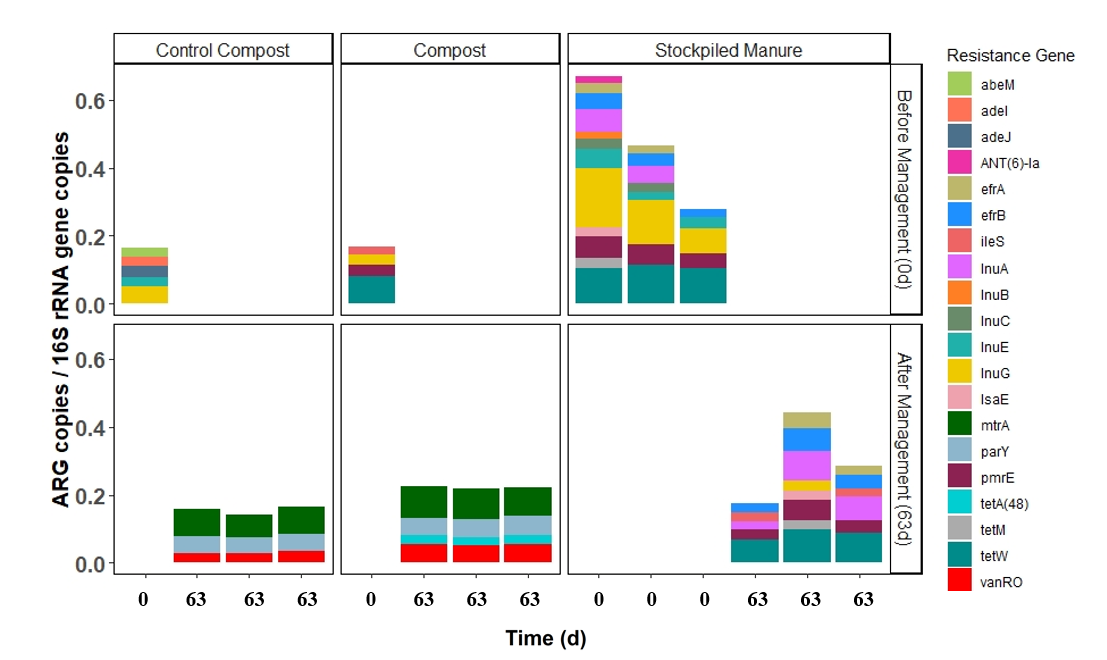


Figure S4. Amendment resistome profiles demonstrated as the top 20 most abundant ARGs before (0d) and after (63d) stockpiling and composting processes represented according to class of antibiotic to which they encode resistance. ARGs were annotated using CARD v2.0.1 via shotgun metagenomic sequencing.


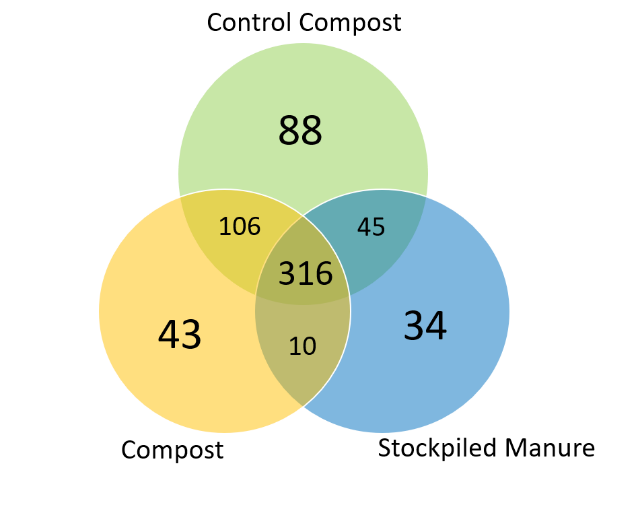


Figure S5. Total number of ARGs annotated via the CARD v2.0.1 database within the three amendment types. Of the 642 ARGs annotated, 316 ARGs were detected within each amendment (i.e. control compost, compost, stockpiled manure.

**
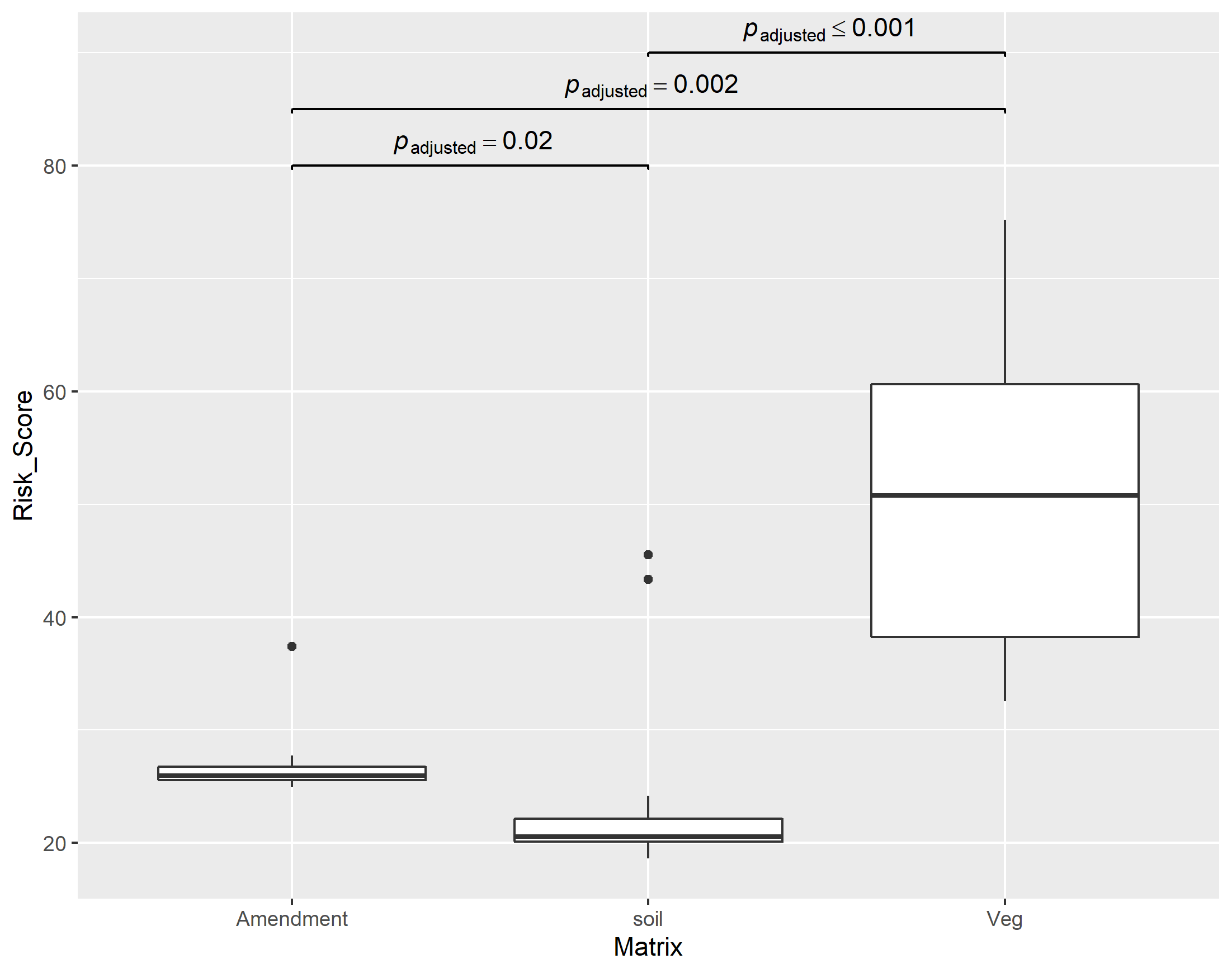
**

Figure S6. Agricultural resistome risk scores compared using the Kruskal-Wallis non-parametric test. Amendment comprises of control compost, compost with antibiotics and stockpiled manure with antibiotic conditions (n=14). Soil comprises of the no amendment control, inorganic fertilizer control, compost with antibiotics, and stockpiled manure with antibiotics samples at the times of background, application, and harvest (n=27). Vegetables comprises of lettuce samples grown in the amended soils at the time of harvest (n=11*), there was out lettuce sample with a risk score of 166 which was considered an outlier for the visualization; further details can be found in Table S2).


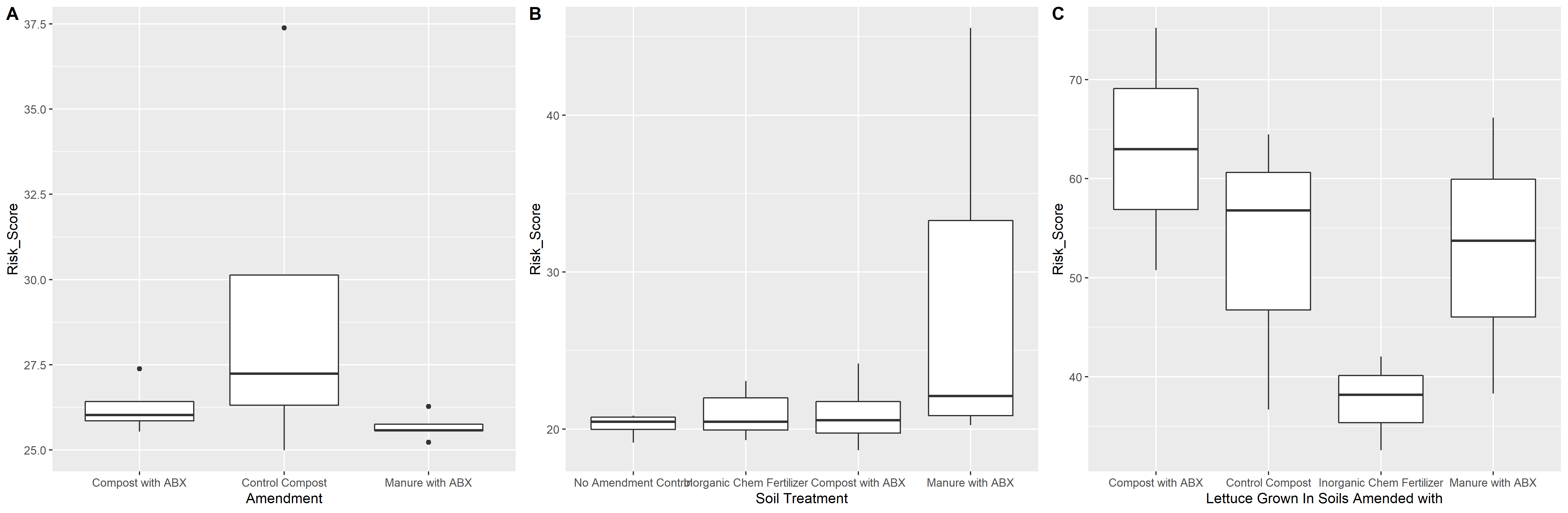


Figure S7. Agricultural resistome risk scores compared using the Kruskal-Wallis non-parametric test at the (A) Amendment, (B) Soil, and (C) Vegetable points along the production chain. Amendment comprises of control compost, compost with antibiotics and stockpiled manure with antibiotic conditions (n=14). Soil comprises of the no amendment control, inorganic fertilizer control, compost with antibiotics, and stockpiled manure with antibiotics samples at the times of background, application, and harvest (n=27). Vegetables comprises of lettuce samples grown in the amended soils at the time of harvest (n=11*), there was out lettuce sample with a risk score of 166 which was considered an outlier for the visualization; further details can be found in Table S3).


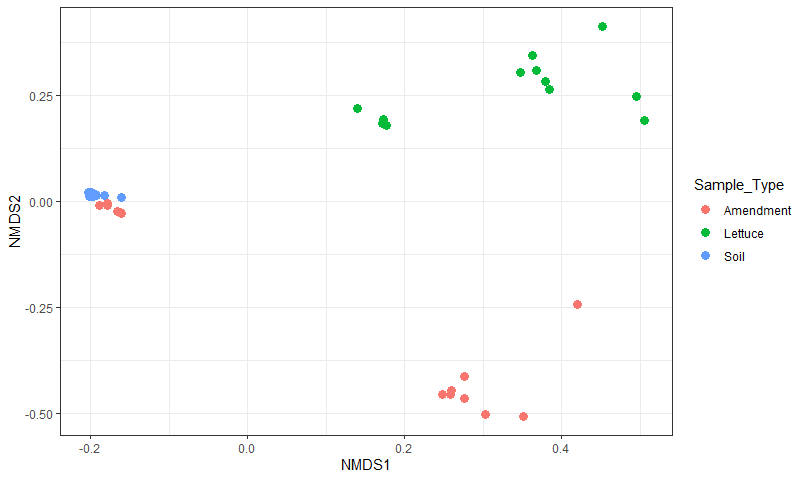


Figure S8. NMDS comparing the taxonomic profiles across the pre-harvest vegetable production chain.
